# Reconstructing directed gene regulatory network by only gene expression data

**DOI:** 10.1186/s12864-016-2791-2

**Published:** 2016-08-18

**Authors:** Lu Zhang, Xi Kang Feng, Yen Kaow Ng, Shuai Cheng Li

**Affiliations:** 1 Department of Computer Science, City University of Hong Kong, Kowloon, Hong Kong; 2 Faculty of Information and Communication Technology, University Tunku Abdul Rahman, Kampar, Perak Malaysia

**Keywords:** Gene regulatory network, Regulatory direction, Important regulators, Gene expression

## Abstract

**Background:**

Accurately identifying gene regulatory network is an important task in understanding in vivo biological activities. The inference of such networks is often accomplished through the use of gene expression data. Many methods have been developed to evaluate gene expression dependencies between transcription factor and its target genes, and some methods also eliminate transitive interactions. The regulatory (or edge) direction is undetermined if the target gene is also a transcription factor. Some methods predict the regulatory directions in the gene regulatory networks by locating the eQTL single nucleotide polymorphism, or by observing the gene expression changes when knocking out/down the candidate transcript factors; regrettably, these additional data are usually unavailable, especially for the samples deriving from human tissues.

**Results:**

In this study, we propose the Context Based Dependency Network (CBDN), a method that is able to infer gene regulatory networks with the regulatory directions from gene expression data only. To determine the regulatory direction, CBDN computes the influence of source to target by evaluating the magnitude changes of expression dependencies between the target gene and the others with conditioning on the source gene. CBDN extends the data processing inequality by involving the dependency direction to distinguish between direct and transitive relationship between genes. We also define two types of important regulators which can influence a majority of the genes in the network directly or indirectly. CBDN can detect both of these two types of important regulators by averaging the influence functions of candidate regulator to the other genes. In our experiments with simulated and real data, even with the regulatory direction taken into account, CBDN outperforms the state-of-the-art approaches for inferring gene regulatory network. CBDN identifies the important regulators in the predicted network: 1. *TYROBP* influences a batch of genes that are related to Alzheimer’s disease; 2. *ZNF329* and *RB1* significantly regulate those ‘mesenchymal’ gene expression signature genes for brain tumors.

**Conclusion:**

By merely leveraging gene expression data, CBDN can efficiently infer the existence of gene-gene interactions as well as their regulatory directions. The constructed networks are helpful in the identification of important regulators for complex diseases.

## Background

Understanding of regulatory mechanisms can help us bridge the gap from genotype to phenotype and enlighten us with more insights on the synthesizing effects of different elements in cells. The advent of high-throughput technology provides us an unprecedent opportunity to construct an atlas of these regulatory mechanisms—the gene regulatory network (GRN)—from which one can study important dynamics such as cell proliferation, differentiation, metabolism, and apoptosis.

GRN is often inferred from gene expression data, which is available in abundance from high-throughput microarray and RNA-Seq. Many computational approaches have been developed to infer the dependencies between transcription factor (TF) and its target genes from expression data. The intuitive method is to consider a regulatory dependency as the correlation of the expressions of the TF-target pair, computed through a measure such as mutual information (MI), Pearson correlation, *etc*. However, the correlations captured within the expression data include the effects of intermediary factors; unless taken into account, they will result in the inclusion of transitive edges in the GRN inferred. To overcome this phenomenon, ARACNE [1], an MI-based method, distinguishes between direct and indirect dependencies by applying data processing inequality. It considers the lowest MI value among any triplet of genes as a transitive edge. CLR (context likelihood of relatedness) [2] presents a framework to consider background noise, which naturally accounts for the transitive effects. The method works on the fact that each gene’s MIs or Pearson correlations with other genes follow the Gaussian distribution. This allows the gene-gene correlations to be expressed as *Z*-scores, thus allowing the comparison of their strengths. Methods based on regression have also been proposed. They incorporate all the genes in a regression model; one as response variable and the others as regressors. Regression-based methods face two difficulties: 1. most of the regressors are not actually independent, hence potentially resulting in erratic regression coefficients for these variables; 2. The model suffers from severe overfitting which necessitates the use of variable selection strategies. A few successful methods have been reported. TIGRESS [3] treats GRN inference as a sparse regression problem and introduce least angle regression in conjunction with stability selection to choose target genes for each TF. GENIE3 [4] performs variables selection based on an ensemble of regression trees (Random Forests or Extra-Trees).

Another kinds of methods are proposed to improve the predicted GRNs by introducing additional information. Considering the heterogeneity of gene expression across different conditions, cMonkey [5] is designed as a bi-clustering algorithm to group genes by assessing their co-expressions and the co-occurrence of their putative *cis*-acting regulatory motifs. The genes grouped in the same cluster are implied to be regulated by the same regulator. Inferelator [6] is developed to infer the GRN for each gene cluster from cMonkey by regression and *L*_1_-norm regularization on gene expression or protein abundance. Recently, Chen et al. [7] demonstrated that involving three dimensional chromatin structure with gene expression can improve the GRN reconstruction. While these methods have relatively good performance in reconstructing GRNs, they are unable to infer regulatory directions.

There have been many attempts at the inference of regulatory directions by introducing external data. The regulatory direction may be determined from *cis* expression single nucleotide polymorphism data, called *cis*-eSNP. The *cis*-eSNPs are thought of as regulatory anchors by influencing the expression of nearby genes. Zhu et al. [8] developed a method called RIMBANET which reconstructs the GRN through a Bayesian network that integrates both gene expression and *cis*-eSNPs. The *cis*-eSNPs determine the regulatory direction with these rules: 1. The genes with *cis*-eSNPs can be the parent of the genes without *cis*-eSNPs; 2. The genes without *cis*-eSNPs cannot be the parent of the genes with *cis*-eSNPs. These strategies have been very successful [9–11]. However, their applicability is limited by the availability of both SNP and gene expression data.

The inference of interaction networks is also actively studied in other fields. Recently, Dror et al. [12] proposed the use of a partial correlation network (PCN) to model the interaction network of a stock market. PCN computes the influence function of stock *A* to *B*, by averaging the influence of *A* in the connectivity between *B* and other stocks. The influence function is asymmetric, so the node with larger influence to the other one is assigned as parent. Their framework has been extended to other fields such as immune system [13] and semantic networks [14]. Nevertheless, there is an obvious drawback in using PCNs for the inference of GRNs: PCNs only determine whether one node is at a higher level than the other. They do not distinguish between the direct and transitive interactions.

**Fig. 1:**
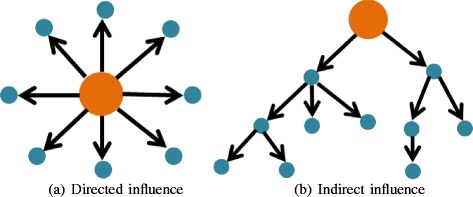
Two types of important regulators with directed influence (**a**) and indirect influence (**b**) to the other genes in the network

Another primary goal of GRN analysis is to identify the important regulator in a network. An important regulator is a gene that influences most of the gene expression signature (GES) genes (e.g. differentially expressed genes) in the network. Carro et al. [15] identified *C/EBPβ* and *STAT3* as important regulators for brain tumor by calculating the overlap between the TF’s targets and ‘mesenchymal’ GES genes based on Fisher’s exact test. TFs were ranked by the number of overlap genes to avoid the influence of the different size of their targets. However, this study only considers the direct influence (Fig. 1(a))of transcription factors to their target genes, the indirect influence (Fig. 1(b)), through transitive genes, are neglected. Zhang et al. [16] developed a method called KDA (key driver analysis) to calculate whether the GES genes are enriched in the targets of regulators by searching *h*-layer neighborhood dynamically or statically with respect to the given directed network. KDA has been extended to search indirect nodes that are influenced by those regulators, but the influence function is qualitative. It ignores the regulatory strength between regulators and their target genes. On the other hand, because the directed network is quantitatively predicted from gene expression data, we cannot regard the interactions as having the same weight.

In this study, we propose a new method, Context Based Dependency Network (CBDN), which introduces the use of an influence function to decide the edge direction. In addition, we show a directed data processing inequality (DDPI), a property of the influence function, which is used to remove transitive interactions in the partial correlation network. Thus each edge predicted by CBDN is both causal and directed, which can be further applied to infer the important regulators quantitatively. The performance of CBDN is compared to a few well-known algorithms, namely ARACNE, CLR, TIGRESS and GENIE3. In the simulation study, CBDN’s result is comparable to the best result of these methods in each situation and proves its outstanding ability to predict regulatory direction. For a realistic test, we point out the *TYROBP*-oriented network which is related to Alzheimer’s disease [17]. In this test, CBDN is superior to other methods in inferring both network structure and regulatory direction. CBDN also successfully infers *TYROBP* as the important regulator by quantitatively considering *TYROBP*’s influences on the other genes. For another real expression data from the patients affected by human brain tumors, CBDN predicts two potential important regulators *ZNF329* and *RB1* whose function are associated with brain tumors. All of these results demonstrate the strength of CBDN in the inference of directed GRNs from gene expression data as well as its potential in predicting important regulators.

## Result

CBDN is designed to construct directed regulatory network by only gene expression data. The computation of CBDN consists of three stages: In the first stage, the influence of each gene to the others is calculated to determine the edge direction. This is done through a partial correlation network constructed from the gene expression data; In the second stage, the transitive interactions are removed by DDPI; In the third stage, the important regulators are inferred by ranking the regulators based on their total influences to the GES genes.

### Determine the edge direction

CBDN infers the regulatory interaction through the influence function. The influence function of gene *A* to gene *B* (denoted as *D*(*A*→*B*)) is calculated by averaging the Pearson correlation changes between gene *B* and the other genes in the network, with or without gene *A*. Notice that the influence function is asymmetric that means *D*(*A*→*B*)≠*D*(*B*→*A*), this phenomenon is adopted to determine the direction of regulatory edge by selecting the genes with larger influence function as the parents. The influence function is derived from partial correlation network, the detailed description can be found in “Methods”.

**Fig. 2:**
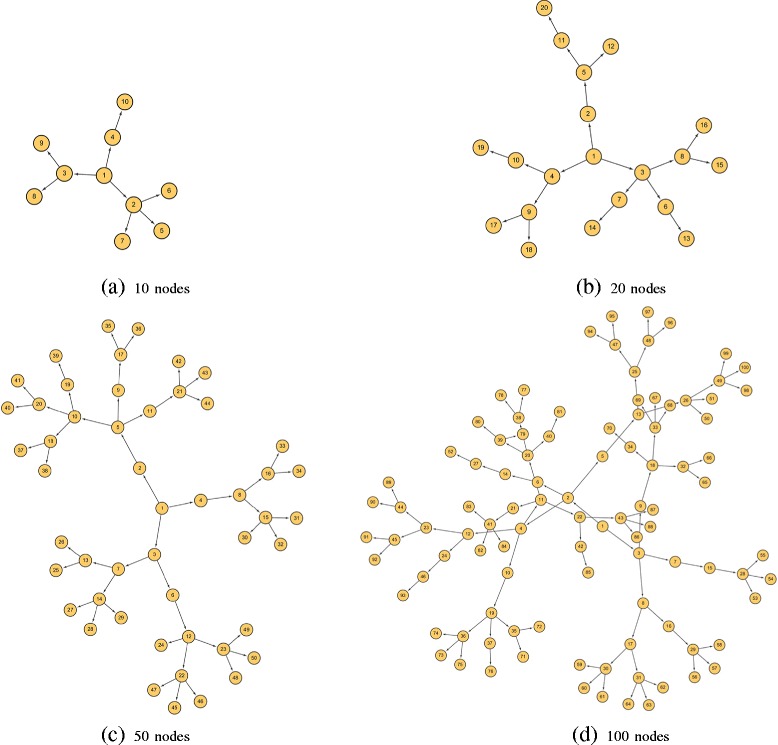
The simulated gene regulatory network structures and edge directions with 10 (**a**), 20 (**b**), 50 (**c**) and 100 (**d**) nodes

Here we give a schematic example based on the simulated GRN structure in Fig. 2(a) to interpret how CBDN determines the edge directionality.

Here, we denote the variable of node *i* as *X*_*i*_. For instance, the direction between *X*_1_ and *X*_4_ is determined by comparing *D*(*X*_1_→*X*_4_) and *D*(*X*_4_→*X*_1_). *X*_4_ merely affects the correlation between *X*_1_ and *X*_10_ (see Methods),

1 $$ D(X_{4}\to X_{1}) =\frac{|Corr(X_{1},X_{10})|}{9}  $$

*Corr*(*X*_*i*_,*X*_*j*_) denotes the Pearson correlation between the two variables *X*_*i*_ and *X*_*j*_. the correlation between *X*_1_ and other variables are not influenced given *X*_4_. When conditioning on *X*_1_, the influences are extended to seven variables (*X*_2_,*X*_3_,*X*_5_,*X*_6_,*X*_7_,*X*_8_ and *X*_9_),

2 $$ D(X_{1}\to X_{4}) =\frac{\Sigma^{2,3,5,6,7,8,9}_{i}|Corr(X_{1},X_{i})|}{9}  $$

The upper bound of *D*(*X*_4_→*X*_1_) (*D*(*X*_4_→*X*_1_)≤1) is smaller than *D*(*X*_1_→*X*_4_) (*D*(*X*_1_→*X*_4_)≤7) in general, so CBDN concludes that *D*(*X*_4_→*X*_1_)≤*D*(*X*_1_→*X*_4_). The edge direction is from *X*_1_ to *X*_4_.

### Directed data processing inequality

**Fig. 3:**
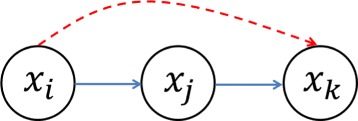
The diagram for how to remove transitive interactions according to DDPI. We assume *X*_*i*_ regulates *X*_*j*_, DDPI is calculated to determine whether *X*_*i*_ directly regulate *X*_*k*_ (red dashed arrow) or through *X*_*j*_ (blue solid arrows)

The influence function described above only determines whether one gene is the parent or child of another gene; it does not provide the regulatory relationship. As an example, the partial correlation network in Fig. 3 identifies *X*_*i*_ as the parent of *X*_*k*_, but does not distinguish whether a transitive relation (*X*_*i*_→*X*_*j*_→*X*_*k*_) exists or not (*X*_*i*_→*X*_*k*_). Data processing inequality (DPI) can be used to remove transitive interactions by assuming the post-processing cannot increase the mutual information. If *X*_*i*_, *X*_*j*_ and *X*_*k*_ form a Markov chain, denoted as *X*_*i*_→*X*_*j*_→*X*_*k*_

3 $$ MI(X_{i}; X_{k})\le MI(X_{i}; X_{j})  $$

which shows that the mutual information between the genes with transitive interaction cannot be greater than direct interaction. This observation has been used in ARACNE to remove transitive interactions for every triplet of genes. Considering the edge direction and the nature of influence function, we propose a directed data processing inequality to show that the influence of a gene which interacts transitively with its target genes cannot be greater than that of a gene which interacts directly, that is

4 $$ D(X_{i}\to X_{k})\le D(X_{j}\to X_{k})  $$

The mathematical proof is straightforward and presented in Methods. We give an example to show how DDPI distinguishes direct (*X*_2_ to *X*_6_) and transitive (*X*_1_ to *X*_6_) interactions in Fig. 2(a). Given *X*_6_, all the other variables are divided into two categories: non-descendent of *X*_2_ and descendent of *X*_2_. The set *U* denotes non-descendent of *X*_2_, including *X*_1_,*X*_2_,*X*_3_,*X*_4_,*X*_8_,*X*_9_,*X*_10_. The descendents of *X*_2_, presented as *V*, consists of *X*_5_ and *X*_7_.

For all the variables in *U*, the influence functions for *X*_1_ (*D*_1_(*X*_1_→*X*_6_)) and *X*_2_ (*D*_1_(*X*_2_→*X*_6_)) are

5 $$ \begin{aligned} D_{1}(X_{1}\to X_{6})&=\frac{\Sigma^{3,4,8,9,10}_{i}|Corr(X_{i},X_{6})|}{6}\\ D_{1}(X_{2}\to X_{6})&=\frac{\Sigma^{1,3,4,8,9,10}_{i}|Corr(X_{i},X_{6})|}{6} \end{aligned}  $$

For all the variables in *V*, the influence functions for *X*_1_ (*D*_2_(*X*_1_→*X*_6_)) and *X*_2_ (*D*_2_(*X*_2_→*X*_6_)) are

6 $$ \begin{aligned} D_{2}(X_{1}\to X_{6})&=0\\ D_{2}(X_{2}\to X_{6})&=\frac{\Sigma^{5,7}_{i}|Corr(X_{i},X_{6})|}{2} \end{aligned}  $$

Then we have

7 $${} \begin{aligned} D_{1}(X_{2}\to X_{6})&>D_{1}(X_{1}\to X_{6})\\ D_{2}(X_{2}\to X_{6})&>D_{2}(X_{1}\to X_{6})\\ D(X_{2}\to X_{6})&=D_{1}(X_{2}\to X_{6})+D_{2}(X_{2}\to X_{6})\\ &>D_{1}(X_{1}\to X_{6})+D_{2}(X_{1}\to X_{6})\\ &=D(X_{1}\to X_{6}) \end{aligned}  $$

*X*_2_ is prefer to be the direct parent of *X*_6_ instead of *X*_1_ according to Eq. 7. Thus the regulatory structure in Fig. 2(a) should be *X*_2_→*X*_6_ rather than *X*_1_→*X*_6_.

To account for the influence of noise, we introduce a tolerance parameter *τ*. A transitive relationship *X*_*j*_→*X*_*k*_ is removed when *D*(*X*_*i*_→*X*_*k*_)−*D*(*X*_*j*_→*X*_*k*_)>*τ*. Otherwise, *X*_*i*_→*X*_*k*_ is removed. Large *τ* implies much more noise exists in the expression data to influence *D*(*X*_*i*_→*X*_*k*_) and *D*(*X*_*j*_→*X*_*k*_).

### Determine the important regulators

The important regulator identified by CBDN is not required to regulate most of the GES genes. Instead, it should have large influence on them, which guarantees such regulator is always on the top level. In this example, *X*_1_ has the largest influence on the other genes in the network and is located on the top level (Methods).

### Simulation

#### Tree structure simulation

In order to explicitly reflect the nature of directed interactions in the gene regulatory network, we simulate a tree structure in which each node has only one parent (except the root) and is merely regulated by its parent (only one arrow from its parent, shown in Fig. 2). In other words, the expression profiles of the descendents are only determined by their parents. The expression profiles for each node were sampled from Gaussian distribution. The joint distribution of the parent and one of its descendent follows bivariate Gaussian distribution with specified covariance and noise. In addition, we mix uniform distributed noise weighted by $\frac {\omega }{\kappa }$ to the simulated expression profiles, where “ *ω*" presents the amount of noise and “ *κ*” denotes the noise level. We set “ *ω*" to a constant (*ω*=3) and change “ *κ*” from 0 to 2 in the simulations. The expression profiles of 10, 20, 50, 100 nodes are simulated, each of them derived from 1000 samples. The network structure and edge direction are shown in Fig. 2.

#### Infer edge direction

**Fig. 4:**
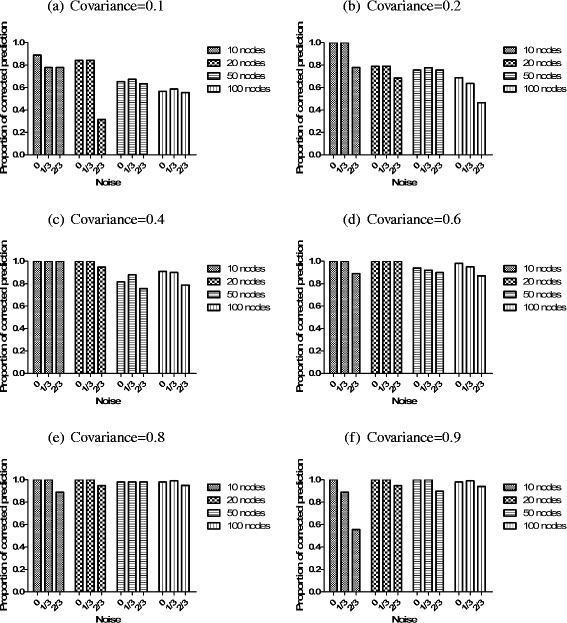
The performance of predicting edge direction by PCN. The increasing covariance spectrum is assigned from 0.1-0.9 in (**a**)-(**f**). Different situations such as the amount of mixed noise and the number of nodes are also evaluated in each subfigure

Based on the partial correlation network, CBDN can predict the interaction edge direction by only gene expression data. In the simulation, we calculate the proportion of edges that are assigned the directions correctly to evaluate the CBDN’s performance. Our simulation results demonstrate excellent performance of CBDN in predicting edge direction (Fig. 4). There are 83.3 % of the simulations (66/72) where at least 60 % of the edges are correctly assigned directions. As the covariance between these nodes increased, the predicted accuracy increases, and reaches optimality when the covariance is above 0.4. The influence of noise is more severe for the networks with small number of nodes (Fig. 4(a), (b) and (f)). The low covariance makes the performance in large networks declined dramatically (Fig. 4(a) and (b)).

#### Compare CBDN with other methods

We evaluate the overall performance of CBDN (including predicted edges and their directions) by comparing it with other famous methods based on a variety of simulated datasets. The true positive rate (TPR) and false positive rate (FPR) are used to plot the receiver operating characteristics (ROC) curve, where $TPR=\frac {TP}{TP+FN}$, $FPR=\frac {FP}{FP+FN}$ (TP:true positive, FN:false negative, FP:false positive). The area under ROC curve (AUC) was applied to evaluate the performance of CBDN. We apply the same tests on four state-of-the-art approaches (ARACNE, CLR, GENIE3 and TIGRESS) for comparison. In Table 1, CBDN’s result is the best when no noise exists. Even with small covariance, CBDN correctly revealed the structure and regulatory orientations (Table 1(a)). When noise is introduced, CBDN’s result remains comparable with the best result in each situation. CBDN worked well in general under medium covariance; large or small covariance make it difficult to distinguish direct and transitive interactions, especially when a large amount of noise is introduced (Table 1). However, our comparison is very conservative here, since the performance of CBDN is evaluated by considering both structure and direction, while the other four methods are evaluated only on the inferred structures. Nevertheless, CBDN achieves sufficiently good performance in reconstructing the directed GRNs. We also simulate tree structures with 20, 50,100 nodes, in which CBDN achieves very similar results as the network with 10 nodes simulation (See Tables 2, 3 and 4).

**Table 1:** Simulation result for 10 nodes tree by comparing CBDN with other methods by AUC

Covariance	ARACNE	CLR	GENIE3	TIGRESS	CBDN
(a) Simulation without					
any noise					
0.1	0.8367	0.8009	0.8765	0.8157	0.8750
0.2	1	1	1	0.8410	1
0.4	1	1	1	0.8502	1
0.6	1	1	1	0.8272	1
0.8	1	1	1	1	1
b) Simulation with 1/3					
random noise					
0.1	0.6304	0.6358	0.5879	0.8107	0.8571
0.2	0.9192	0.9846	0.9884	0.8162	1
0.4	1	1	1	0.8327	1
0.6	1	1	1	0.8557	1
0.8	1	1	0.9985	0.8338	1
(c) Simulation with 2/3					
random noise					
0.1	0.6904	0.6172	0.6813	0.6241	0.8571
0.2	0.6889	0.8086	0.8480	0.8309	1
0.4	0.9531	0.9599	0.9437	0.8428	1
0.6	1	1	0.9931	0.8424	0.8750
0.8	0.9333	0.9907	0.9807	0.8058	0.8750

**Table 2:** Simulation result for 20 nodes tree by comparing CBDN with other methods by AUC

Covariance	ARACNE	CLR	GENIE3	TIGRESS	CBDN
(a) Simulation without					
any noise					
0.1	0.8775	0.9332	0.9747	0.7916	0.9306
0.2	0.9961	0.9963	0.9985	0.8034	1
0.4	1	1	1	0.8245	1
0.6	1	1	1	0.7975	1
0.8	1	1	1	0.8015	1
(b) Simulation with					
1/3 random noise					
0.1	0.7261	0.8864	0.8369	0.7812	0.8269
0.2	0.9166	0.9836	0.9877	0.7940	0.9286
0.4	1	1	1	0.8249	1
0.6	1	1	1	0.7845	1
0.8	1	1	0.9996	0.8387	1
(c) Simulation with 2/3					
random noise					
0.1	0.6364	0.5499	0.5748	0.5848	0.7500
0.2	0.7797	0.8680	0.9146	0.7735	0.8462
0.4	0.9825	0.9905	0.9988	0.8126	1
0.6	0.9977	1	0.9994	0.8465	0.9000
0.8	0.8804	0.9920	0.9911	0.8146	1

**Table 3:** Simulation result for 50 nodes tree by comparing CBDN with other methods by AUC

Covariance	ARACNE	CLR	GENIE3	TIGRESS	CBDN
(a) Simulation without					
any noise					
0.1	0.7643	0.8991	0.9225	0.8562	0.8646
0.2	0.9988	0.9997	0.9999	0.8352	0.9762
0.4	1	1	1	0.8448	0.9286
0.6	1	1	1	0.8483	0.9902
0.8	1	1	1	0.8470	1
(b) Simulation with					
1/3 random noise					
0.1	0.7018	0.7831	0.8208	0.8151	0.7561
0.2	0.9617	0.9936	0.9985	0.8409	0.9748
0.4	1	0.9999	1	0.8738	0.9688
0.6	1	1	1	0.9032	1
0.8	1	0.9994	0.9998	0.9300	1
(c) Simulation with 2/3					
random noise					
0.1	0.6266	0.5486	0.6385	0.6712	0.7561
0.2	0.6196	0.7746	0.8675	0.8139	0.9625
0.4	0.9893	0.9967	0.9991	0.8673	0.8600
0.6	0.9948	0.9982	0.9982	0.8828	0.9697
0.8	0.9286	0.9943	0.9942	0.9043	1

**Table 4:** Simulation result for 100 nodes tree by comparing CBDN with other methods by AUC

Covariance	ARACNE	CLR	GENIE3	TIGRESS	CBDN
(a) Simulation without					
any noise					
0.1	0.7445	0.8674	0.9388	0.8394	0.9804
0.2	0.9976	0.9995	1	0.8632	0.9231
0.4	1	1	1	0.8676	0.9792
0.6	1	1	1	0.8872	1
0.8	1	1	0.8426	0.9018	1
(b) Simulation with					
1/3 random noise					
0.1	0.6929	0.7572	0.8303	0.7765	0.8333
0.2	0.9561	0.9915	0.9992	0.8615	0.9894
0.4	1	1	1	0.8745	0.9875
0.6	1	1	1	0.9071	0.9905
0.8	1	0.9992	1	0.9511	0.9965
(c) Simulation with 2/3					
random noise					
0.1	0.4874	0.6362	0.6480	0.6547	0.9756
0.2	0.7527	0.8294	0.8867	0.8169	0.9794
0.4	0.9737	0.9871	0.9976	0.8843	0.9938
0.6	0.9990	0.9996	0.9998	0.9237	0.9907
0.8	0.9520	0.9973	0.9979	0.9123	0.9965

#### Infer important regulators

**Fig. 5:**
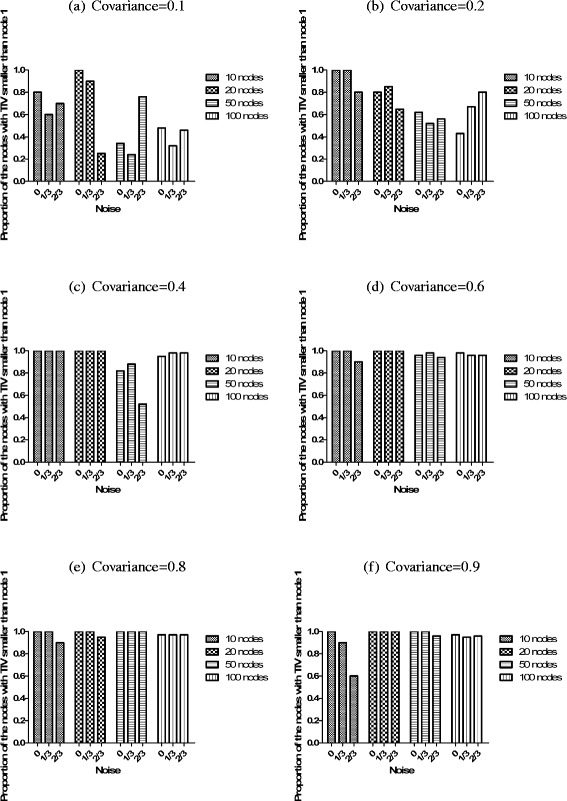
The performance of predicting important regulator by DDPI. The increasing covariance spectrum is assigned from 0.1-0.9 in (**a**)-(**f**). Different situations such as the amount of mixed noise and the number of nodes are also evaluated in each subfigure

From the network structure for simulation (Fig. 2), the confirmed important regulator is node 1, which is the parent of all the other nodes in the network. Here, we calculate the proportion of those nodes in the network, whose total influence value *TIV* (Methods) is smaller than the *TIV* for node 1, to evaluate the inference ability of CBDN. From Fig. 5(a) and (b), we see that smaller networks are in general inferred more accurately, while the effects of noise is unpredictable. For example, for the 50 nodes network in Fig. 5(a), the case with 2/3 noise applied is better predicted than the cases with smaller noise. The important regulator prediction is unstable and unbelievable in the network with weak correlation. The proportion tends to one when the covariance is larger than 0.6 and the nodes in the network are larger than 20 (Fig. 5(d), (e) and (f)), which suggest that the inference is quite reliable for above medium covariance.

### Real data

**Fig. 6:**
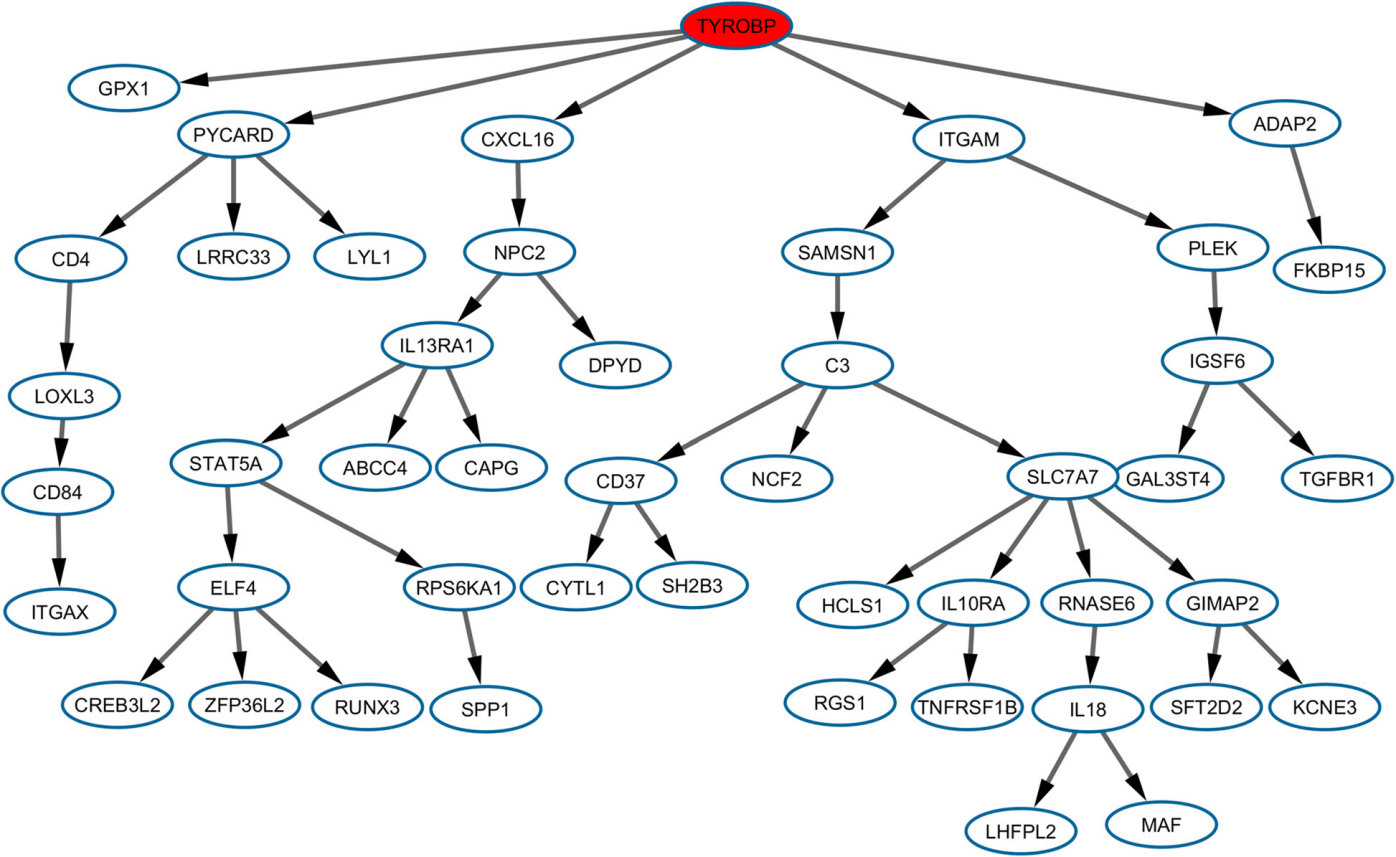
The network structure for the *TYROBP* oriented regulatory network for Alzheimer’s disease

For this test, we download the processed expression data from GEO [18] (GSE44770), which is from dorsolateral prefrontal cortex of human brains. The expression data include 230 tissues from the individuals with or without Alzheimer’s disease. The negative expression values are considered missing values because of their low intensities compared to background noise. We impute these missing values with the average positive expression values across all the samples of the same gene. Using gene expression and *cis*-eSNPs data, Zhang et al. [17] had earlier found the disease-related network to be regulated by *TYROBP*. In addition, loss-of-function-mutations were recognized in *TYROBP* in Finnish and Japanese patients affected by presenile dementia with bone cysts [19]. Zhang et al. also overexpressed either full-length or a truncated version of *TYROBP* in microglia cells from mouse embryonic stem cells to confirm the structure and direction of the regulatory network (Fig. 6). From the *TYROBP* regulatory network, we choose 47 GES genes, the expressions of which are altered when *TYROBP* is overexpressed and captured by microarray data, multiple probes designed for the same gene are combined by averaging their expression values.

**Fig. 7:**
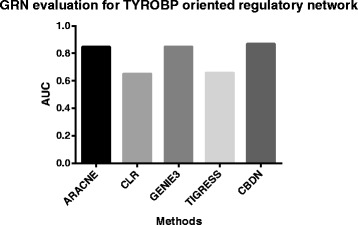
The performance of different methods for predicting *TYROBP* oriented regulatory network

**Fig. 8:**
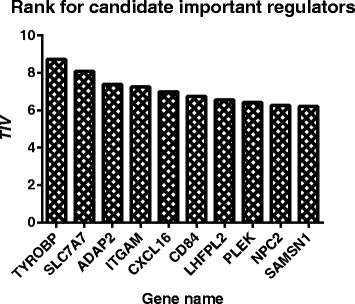
The top ten genes with the largest *TIV* values for Alzheimer’s disease

This dataset is then used as the input for ARACNE, CLR, GENIE3, TIGRESS, and CBDN. The results are compared with the true network structure and edge directions from mouse embryonic stem cells experiment. Figure 7 demonstrates the AUC scores for the five methods. CBDN achieves the best performance, which is 2 *%* higher than the second best result from GENIE3. To evaluate the capability of CBDN in predicting the regulatory direction and important regulator, we assume all the genes to be potential regulators and ranked them based on *TIV*. If one gene is assessed as a regulators, other genes are assumed to be GES genes. Figure 8 lists the top 10 genes with the largest *TIV*, only the values of *TYROBP* and *SLC7A7* are above 8, the validate important regulator *TYROBP* is ranked at the top. *SLC7A7* regulates eleven GES genes (*HCLS1*, *IL10RA*, *RNASE6*, *GIMAP2*, *RGS1*, *TNFRSF1B*, *IL18*, *SFT2D2*, *KCNE3*, *LHFPL2* and *MAF*) and may be another candidate regulator and required to be validated in the future.

**Fig. 9:**
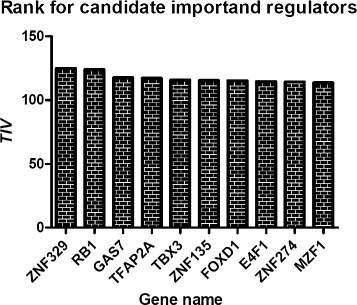
The top ten genes with the largest *TIV* values for brain tumors

For another experiment, we download the expression data for brain tumors (GSE19114) and pre-process them as for Alzheimer’s disease. Eventually, we choose 132 ’mesenchymal’ gene expression signature (MGES) genes and 883 TFs from Supplementary Tables 1 and 2 from the original paper [15]. Both MGES genes and TFs are combined together to calculate *TIV* for each TFs, because we are also required to consider the regulatory relationships between TFs. We are unable to identify the two key regulators (*STAT3* and *C/EBPβ*) described in the original papers from the top *TIV* ranked TFs (Fig. 9), because we adopt different definitions and inherent characteristics of important regulators. The top two TFs, *ZNF329* and *RB1* with *TIV*s exceed 120, are selected as new candidate important regulators. The relationship between *ZNF329* and brain tumors is still unclear, but zinc finger protein family has been proved to be associated with brain tumor. Zhao et al. [20] identified *ZNF325* as a transcription repressor in MAPK/ERK signaling pathway. Recently, Das et al. [21] made a comprehensive review to clarify the relationship between MAPK/ERK signaling pathway and brain tumors and how can one inhibit this pathway to treat paediatric brain tumors. *RB1* gene is the most important cell cycle regulatory genes and the first reported human tumor suppressor gene. It has been identified to be related with a variety of human cancers including brain tumors [22]. Mathivanan et al. found loss of heterozygosity and deregulated expression of *RB1* in human brain tumors [23].

## Discussion

In this paper, we propose a new computational method called Context Based Dependency Network (CBDN), which constructs directed GRNs from only gene expression data. This provides us an opportunity to gain deeper insights from the readily available gene expression data that we have accumulated for years in databases such as GEO. Although gene expression data can reflect the gene-gene interactions in GRN, there are still three limitations that must be addressed. First, the transcription factors prefer to act together as a protein complex rather than individually. The protein complex may be blocked or inactivated, for reasons such as incorrect folding, being restricted in the nucleus or inactivated by the phosphorylation or other modifications, *etc.*, even if its transcribed mRNA has high expression level. Second, the expression of TF and TF binding are time-dependent. Because the time delay exists between transcription and translation, high mRNA expression level does not imply a simultaneous high in protein abundance. Third, even when TFs are bound to their target genes, they may demonstrate different effects because of their three dimensional distances and histone modification.

The probes with low florescence signals are impossible to be distinguished from background noise. CBDN treats them as missing values and imputes them by the average value of the other samples. We have further tested other gene expression imputation methods such as the impute package from Bioconductor or BPCA [24], the reconstructed GRN seems stable and consistence. In the future, some noise filtering methods should be incorporated in CBDN such as described in [25, 26].

The performances of CBDN are underestimated for both simulated and real expression data. Except CBDN, the true positive results are defined as the interactions exist in both predictions and ground truth, which neglect the edge direction. For CBDN, both of the interactions and directions are taken into consideration for evaluating its performance. Even though only 2 % of AUC is improved in *TYROBP*oriented GRN inference, the result is more powerful and useful since they incorporate edge directions. The performance of CBDN is significantly better than other methods in some situations such as Table 1(c) with covariance =0.1, but most of the time CBDN is only slightly better or comparable with other methods.

We believe that CBDN will be invaluable to biomedical studies by transcriptome sequencing, where there is a need for the identification of important regulators. Such studies used to be limited by the availability of SNP data to anchor regulatory directions. However, CBDN may be able to infer such important regulators from gene expression data alone, as it identifies the important regulator *TYROBP* in Alzheimer’s disease. Because CBDN uses new concept of important regulators, it can also help us get new findings which may be neglected by the previous approaches.

This paper also contributes to mathematics in the form of an inequality for directed data processing (DDPI) which naturally extends the data processing inequality for mutual information. DDPI is applied to remove transitive interactions in CBDN.

In the future CBDN should be extended to predict bi-directed interactions which are quite common in nature. By incorporating external data, we hope to use it to tackle the situations where more than one TFs co-regulate a gene simultaneously.

## Conclusion

The reconstruction of gene regulatory network has been actively researched in the past decade, many methods have been designed to achieve this using only high-throughput gene expression data. However, the edge direction is usually unknown and seems hard to be determined by only gene expression data. Even when the directions can be affirmed, the available approaches is unable to remove transitive interactions from directed network. Here, we propose a novel method CBDN, which can reconstruct direct gene regulatory network by only gene expression data. CBDN first constructs an asymmetric partial correlation network to determine the two influence functions for each pair of genes and determine the edge direction between them. DDPI extends data processing inequality applied in directed network to remove transitive interactions. By aggregating the influence function to all the nodes in the network, the total influence value is calculated to assess whether the node is an important regulator. For both simulation and real data test, CBDN demonstrated superior performance compared to other available methods in reconstructing directed gene regulatory network. It also successfully identified the important regulators for Alzheimer’s disease and brain tumors.

## Methods

### Partial correlation network

In CBDN, a partial correlation network is first constructed to compute the influence of each node to the others. Interaction directions are resolved by choosing the node with a larger influence as the parent. The influence of gene *A* to gene *B* is calculated by averaging the difference between the shortest topological paths of gene *B* to other genes with or without gene *A*. We assume the input data is an *m*×*n* matrix, *E*=(*e*_*i*,*j*_)_*m*×*n*_, where each row *i* (denoted *E*_*i*,∙_) represents a sample; that is, one expression value per gene; and each column *j* (denoted *E*_∙,*j*_) represents the expression values of a gene across all the samples.

The partial correlation between *X*_*i*_ and *X*_*k*_ given *X*_*j*_ is calculated as

8 $${} \begin{aligned} PC(X_{i},X_{k}|X_{j})=\frac{Corr(X_{i},X_{k})-Corr(X_{i},X_{j})Corr(X_{k},X_{j})}{\sqrt{[1-Corr(X_{i},X_{j})^{2}][1-Corr(X_{k},X_{j})^{2}]}} \end{aligned}  $$

Where *Corr*(*X*_*i*_,*X*_*j*_) is the Pearson correlation between two genes *X*_*i*_ and *X*_*j*_. The influence of gene *X*_*j*_ for the correlation between *X*_*i*_ and *X*_*k*_ (*k*≠*j*) is defined as the difference between *Corr*(*X*_*i*_,*X*_*j*_) and *PC*(*X*_*i*_,*X*_*k*_|*X*_*j*_),

9 $$ d(X_{i},X_{k}|X_{j})=Corr(X_{i},X_{k})-PC(X_{i},X_{k}|X_{j})  $$

The influence of gene *X*_*j*_ to *X*_*i*_, *D*(*X*_*j*_→*X*_*i*_) is the average *d*(*X*_*i*_,*X*_*k*_|*X*_*j*_) across all the gene *X*_*k*_,

10 $$ D(X_{j}\to X_{i})=\frac{1}{n-1}\Sigma_{k\ne j}^{n-1}|d(X_{i},X_{k}|X_{j})|  $$

CBDN assumes no two-gene cyclic regulation in the network, so we remove the interaction *X*_*i*_→*X*_*j*_ if *D*(*X*_*i*_→*X*_*j*_)<*D*(*X*_*j*_→*X*_*i*_), and vice versa.

### Proof for directed data processing inequality

In the directed GRN, we assume three genes (*X*_*i*_, *X*_*j*_ and *X*_*k*_) form a Markov chain (*X*_*i*_→*X*_*j*_→*X*_*k*_), the other genes are separated into two categories: non-descendents of *X*_*i*_ (*U*={*X*_*m*_⋯*X*_*n*_}) and descendents of *X*_*i*_ (*V*={*X*_*p*_⋯*X*_*a*_}). For the elements in *U*,

11 $$ D_{1}(X_{i}\to X_{k})=\frac{1}{|U|}\Sigma_{t\ne i}^{|U|}|d(X_{k},X_{t}|X_{i})|  $$

12 $$ D_{1}(X_{j}\to X_{k})=\frac{1}{|U|}\Sigma_{t\ne j}^{|U|}|d(X_{k},X_{t}|X_{j})|  $$

Based on Eq. 9, *X*_*k*_ is conditionally independent with the elements in *U* given *X*_*i*_ or *X*_*j*_, thus we have *PC*(*X*_*k*_,*X*_*t*_|*X*_*j*_)=*PC*(*X*_*k*_,*X*_*t*_|*X*_*i*_)=0, |*d*(*X*_*k*_,*X*_*t*_|*X*_*i*_)|=|*d*(*X*_*k*_,*X*_*t*_|*X*_*j*_)|=|*Corr*(*X*_*k*_,*X*_*t*_)|,∀*t*∈*U*. For the genes in *U*, *X*_*i*_ and *X*_*j*_ have the same influence to *X*_*k*_, *D*_1_(*X*_*i*_→*X*_*k*_)=*D*_1_(*X*_*j*_→*X*_*k*_).

For the elements in *V*

13 $$ D_{2}(X_{i}\to X_{k})=\frac{1}{|V|}\Sigma_{t\ne i}^{|V|}|d(X_{k},X_{t}|X_{i})|  $$

14 $$ D_{2}(X_{j}\to X_{k})=\frac{1}{|V|}\Sigma_{t\ne j}^{|V|}|d(X_{k},X_{t}|X_{j})|  $$

Because *X*_*k*_ is the direct descendent of *X*_*j*_, *X*_*k*_ is independent with other genes in *V* given *X*_*j*_ (*PC*(*X*_*k*_,*X*_*t*_|*X*_*j*_)=0,*d*(*X*_*k*_,*X*_*t*_|*X*_*j*_)=|*Corr*(*X*_*k*_,*X*_*t*_)|≥0,∀*t*∈*V*). The correlations between *X*_*k*_ and the other genes in *V* do not change when given *X*_*i*_, so |*d*(*X*_*k*_,*X*_*t*_|*X*_*i*_)|=0,∀*t*∈*V*. We conclude that *D*_2_(*X*_*i*_→*X*_*k*_)=0 and *D*_2_(*X*_*j*_→*X*_*k*_)≥0

15 $$ \begin{aligned} D(X_{i}\to X_{k})&=D_{1}(X_{i}\to X_{k})+D_{2}(X_{i}\to X_{k})\\ &\le D_{1}(X_{j}\to X_{k})+D_{2}(X_{j}\to X_{k})\\ &=D(X_{j}\to X_{k})\\ \end{aligned}  $$

### Determine the important regulators

We propose a new method to identify the important regulators in a quantitative way. Assume the genes with gene expression signature (GES) (eg. differentially expressed genes) are *X*_*s*1_,*X*_*s*2_,…,*X*_*sn*_, the total influence value (*TIV*) of gene *X*_*i*_ is $TIV(X_{i})=\Sigma _{t=1}^{n} D(X_{i}\to X_{st})$. Regulators are ranked by their *TIV*s.
